# Long-term outcome in survivors of neonatal tetanus following specialist intensive care in Vietnam

**DOI:** 10.1186/s12879-017-2748-3

**Published:** 2017-09-25

**Authors:** Huynh T. Trieu, Nguyen Thi Kim Anh, Huynh Ngoc Thien Vuong, T. T. M. Dao, Nguyen Thi Xuan Hoa, Vo Ngoc Cat Tuong, Pham Tam Dinh, Bridget Wills, Phan Tu Qui, Le Van Tan, Lam Minh Yen, Saraswathy Sabanathan, Catherine Louise Thwaites

**Affiliations:** 1 Hospital for Tropical Diseases, 764 Vo Van Kiet, Ho Chi Minh City, Vietnam; 2 Oxford University Clinical Research Unit, Ho Chi Minh City, Vietnam; 3 Centre for Tropical Medicine and Global Health, University of Oxford, Oxford, UK

**Keywords:** Neonatal tetanus, Development, Outcome

## Abstract

**Background:**

Neonatal tetanus continues to occur in many resource-limited settings but there are few data regarding long-term neurological outcome from the disease, especially in settings with critical care facilities.

**Methods:**

We assessed long-term outcome following neonatal tetanus in infants treated in a pediatric intensive care unit in southern Vietnam. Neurological and neurodevelopmental testing was performed in 17 survivors of neonatal tetanus and 18 control children from the same communities using tools previously validated in Vietnamese children.

**Results:**

The median age of children assessed was 36 months. Eight neonatal tetanus survivors and 9 community control cases aged < 42 months were tested using the Bayley III Scales of Infant and Toddler Development (Bayley III-VN) and 8 neonatal tetanus survivors and 9 community controls aged ≥42 months were tested using the Movement Assessment Battery for Children. No significant reductions in growth indices or neurodevelopmental scores were shown in survivors of neonatal tetanus compared to controls although there was a trend towards lower scores in neonatal tetanus survivors. Neurological examination was normal in all children except for two neonatal tetanus survivors with perceptive deafness and one child with mild gross motor abnormality. Neonatal tetanus survivors who had expienced severe disease (Ablett grade ≥ 3) had lower total Bayley III-VN scores than those with mild disease (15 (IQR 14–18) vs 24 (IQR 19–27), *p* = 0.05) with a significantly lower cognitive domain score (3 (IQR 2–6) severe disease vs 7 (IQR 7–8) mild disease, *p* = 0.02).

**Conclusions:**

Neonatal tetanus is associated with long-term sequelae in those with severe disease. In view of these findings, prevention of neonatal tetanus should remain a priority.

## Background

Neonatal tetanus is preventable by maternal vaccination and good birth hygiene. Elimination is the central objective of a highly successful initiative led by the World Health Organization (WHO) and its partners, now in its 3rd decade. The initiative has achieved considerable success with 40 countries having officially eliminated the disease. Nevertheless, as elimination is defined as less than one case per thousand live births in every district of a country, cases of neonatal tetanus continue to occur in many resource-limited settings [1, 2].

Mortality rates from neonatal tetanus vary widely, largely according to facilities available for treatment, particularly mechanical ventilation [3, 4]. Recently we reported improved outcome following the introduction of advanced critical care facilities, including use of invasive blood pressure monitoring and early treatment of autonomic nervous system dysfunction [1]. However although short-term outcome of these infants has improved, prolonged periods of mechanical ventilation may be necessary and infants are thus at risk of many of the complications of critical illness [1]. The long-term consequences of both neonatal tetanus and of managment–related complications remain poorly characterized, especially in settings where in-hospital mortality is low.

Until about 40 years ago neurological outcome from neonatal tetanus was considered to be normal in those who survive. However, studies from the 1980’s suggested poor outcomes in some survivors [5, 6]. Teknezki et al. reported ‘appreciable handicap’ in 4 of 38 survivors of neonatal tetanus seen at least 3 years after hospital discharge, including low IQ score, cerebral palsy and behavioural problems [5]. Anlar followed up 20 neonatal tetanus survivors and reported height or weight measurements were below 5th centiles for age in more than half, with neurodevelopmental impairment occurring in 8 cases [6]. A more recent study from Africa described 123 neonates with tetanus managed without mechanical ventilation; 84 (68%) died in hospital, while among the 39 discharged home, four died within 4 days after leaving the hospital and two more infants died at 3 and 6 months of age. In surviving children assessed 3 to 7 years later significant reductions in head circumference and hand-eye coordination were detected, in addition to non-significant reductions in gross motor and speech and language capabilities [7].

Understanding the long-term consequences of neonatal tetanus is needed to adequately evaluate management strategies, and assess the public health burden and cost-benefit of new interventions or established prevention programs. As critical care facilities improve throughout the world it is necessary to justify resource allocation and ascertain whether improvements in mortality occur at the expense of serious long-term disability, especially in settings where there is little provision for rehabilitation. Now, as many low and middle income countries begin to address the Sustainable Development Goals, the importance of identifying of children at risk of poor development is increasingly recognized [8].

In this study, we assessed the long-term outcomes of survivors of neonatal tetanus 2–5 years after treatment in a specialist pediatric intensive care unit, offering insight not only about neonatal tetanus specifically, but also providing a broader perspective on long-term outcomes after critical illness in a resource-limited setting.

## Methods

The study was approved by the Ethics Committee of the Hospital for Tropical Diseases, Ho Chi Minh City.

Contact details of all surviving infants admitted to the Pediatric Intensive Care Unit at the Hospital for Tropical Diseases with a diagnosis of neonatal tetanus between January 2010 and December 2014 were retrieved from the hospital database, relying on the discharge ICD code A33. Attempts were made to contact all families of cases surviving to hospital discharge by telephone or through local health centers. If families agreed to participate in the study, case records of infants were retrieved from storage then clinical and demographic data from the period of hospitalization were recorded using a standard case record form and entered onto a secure database. All cases of neonatal tetanus were diagnosed clinically according to the World Health Organization criteria [9]. Doctors making this diagnosis were familiar with neonatal tetanus and no cases were felt to be unusual or unlikely to be neonatal tetanus. Disease severity was assessed using modified Ablett score [10].

Neurological and developmental assessment of neonatal tetanus survivors and community controls were undertaken by the same staff, and performed under similar conditions. All the cases came from separate communes across remote provinces in Southern Viet Nam, where ethnic minority groups rely on susbsistence farming. Community controls were selected by local health-care staff from the same villages as cases and matched for ethnicity, age sex and socio-economic status. Age was matched to within 3 months to ensure Bayley III-VN assessment ‘start points’ were the same as cases. Neurological assessment was performed by a specialist pediatrician. For the developmental assessment a locally adapted Bayley Infant Scale of Neurodevelopment-III (Bayley III-VN) was used for children under 42 months of age, with the Movement Assessment Battery for Children used for older children [11, 12]. All tests were performed by five specially-trained pediatric psychologists with extensive experience using these tools, either in the local health centre or in the child’s own home to provide the reassurance of a familiar environment. Given the remote locations in which all the children live, sophisticated supplementary examination (eg audiological assessment) was not possible. Blinding of assessors was not feasible since some of the staff had managed individual cases when they were in hospital.

The Bayley III-VN comprises a standard series of measurements assessing cognitive, fine motor, and gross motor domains. A series of tasks evaluating these domains are conducted with ‘start points’ depending on age. Raw scores are converted to scale scores and then to composite scores. Similarly the Movement Assessment Battery for Children assesses manual dexterity, ball skills and static and dynamic balance, generating a series of scores that are then normalized and converted to a composite score. These neurodevelopment assessment tools are the only international recognized systems to have been translated and validated in a Vietnamese population, and were chosen to best assess previously highlighted deficits following neonatal tetanus. However, due to the variety of languages spoken by the remote ethinic minority communities in which neonatal tetanus commonly occurs, expressive and receptive language components of the Bayley III-VN oculd not be assessed. To compare results for the cognitive, fine motor and gross motor domains with those of the more general population of Vietnamese children, the number of children with raw domain scores 2 or more standard deviations below the means for healthy Vietnamese children was calculated. However, for the Movement Assessment Battery for Children no population mean scores for manual dexterity, ball skills and static and dynamic balance were available for comparison.

Statistical analysis was carried out using Stata Statistical Software: StataCorp. 2003: Release 8. College Station, TX: StataCorp LP. All continuous data are described as median and interquartile range (IQR), with comparison of medians performed using the Mann-Whitney U test. Tests of proportion for categorical variables were carried out using Fisher’s exact test. *P* values ≤0.05 were taken to be significant.

## Results

A total of 32 neonates with tetanus were admitted to the pediatric intensive care unit at the Hospital for Tropical Diseases between 2010 and 2014, of whom 29 survived to hospital discharge. Two children lived in extremely remote locations which could not be reached for assessment and were therefore excluded, and two further infants were found to have died following discharge from hospital. One had been discharged from hospital against medical advice and died within 3 days, while the other died approximately 2 years after discharge and was reported by local healthcare workers to have died of pneumonia. Of the remainder, the families of 17 surviving neonatal tetanus cases were successfully contacted and agreed for their child to participate in the study. Local healthcare workers identified a total of 18 controls living in the same communities as the surviving cases. Thus a total of 17 neonatal tetanus survivors (10 males and 7 females) and 18 controls (10 males and 8 females) were assessed.

The median age of neonatal tetanus survivors was 36 months (IQR 30–59) and of controls 40.5 months (IQR 35–63) (*p* = 0.41). Survivors of neonatal tetanus were generally smaller than controls although no differences were statistically significant (Table 1). Neurological examination was normal in 18/18 controls and 14/17 (%) of cases (*p* = 0.051). Among the three cases with abnormal neurological examination, two cases were found to have hearing impairment, one case had mild gross motor abnormality. No other neurological abnormalities were detected; notably no epilepsy or cerebral palsy.

**Table 1:** Median (IQR) Height, weight and head circumference of survivors of neonatal tetanus (cases) and controls

	Cases (*n* = 17)	Controls (*n* = 18)	
Height (m)	0.9 (0.85–1.02)	11.8 (10.8–14)	*p* = 0.09
Weight (kg)	13 (12–16)	13 (12–16)	*p* = 0.06
Head circumference (cm)	47 (46–48)	47.75 (47–48)	*p* = 0.12

**Fig. 1:**
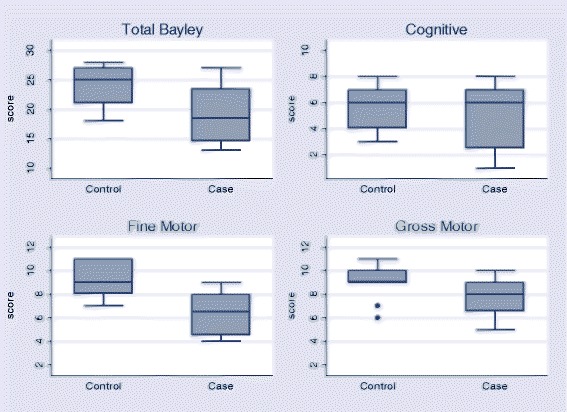
Assessment using Bayley III-VN Scales of Infant and Toddler Development: total scores (summated cognitive, fine motor and gross motor) and individual components for neonatal tetanus survivors (cases) and controls

Seventeen children (8 cases and 9 controls) underwent neurodevelopmental assessment using the Bayley III-VN. One child (a neonatal tetanus survivor) was unable to carry out the assessment due to severe hearing impairment. There was no significant difference in total scores between neonatal tetanus survivors and controls (survivors median score 18.5 (14.5–23.5) compared with controls median score 25 (21–27), *p* = 0.07). There was a trend towards lower component domain scores in cases compared to controls (Fig. 1) but differences were not statistically significant. There was no difference between number of neonatal tetanus survivors or controls with cognitive or gross motor domain scores 2 or more standard deviations below normal for age-matched Vietnamese children (*p* = 0.58 and 0.45 respectively). However 6/8 (75%) neonatal tetanus survivors, but no controls (0/9) had fine motor scores less than two standard deviations below the population mean (*p* < 0.01).

**Fig. 2:**
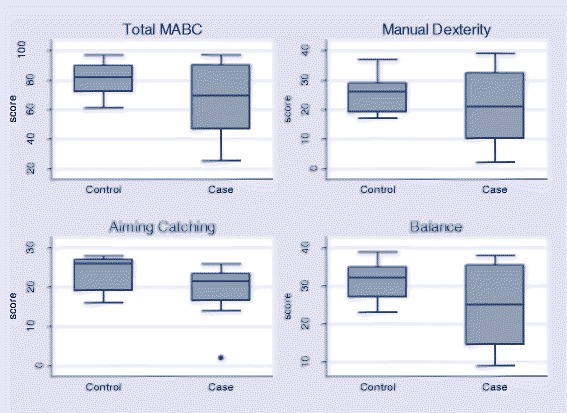
Movement Assessment Battery for Children scores of cases neonatal tetanus survivors (cases) and controls showing total score (MABC: summated manual dexterity, aiming catching and balance) and individual components

Seventeen older children (8 cases and 9 controls) were assessed with the Movement Assessment Battery for Children tool. Again the composite scores showed that, although there was a trend towards lower scores in cases compared to controls, there were no statistically significant differences (Fig. 2).

From review of hospital records, a total of 12 cases were classified as having severe disease (Modified Ablett scores III or IV) while 5 cases had mild disease (Ablett scores I or II, not requiring mechanical ventilation). Baseline clinical data for these infants are given in Table 2. Several known prognostic indicators – birth weight, incubation period, and day of illness at presentation – were significantly different between the mild and severe groups (Table 2) [13].

**Table 2:** Comparison of prognostic indicators of neonatal tetanus according to severity

	Severe (*n* = 12)	Mild (*n* = 5)	
Birth Weight (g)	2500(2400–3000)^a^	3100 (3000–3600)	*p* = 0.04
Day of illness at hospital presentation	2.5 (2–3.5)	5 (5–5)	p = 0.02
Incubation period (days)	7 (7–10)	6 (5–7)	p = 0.04

^a^n = 11

**Fig. 3:**
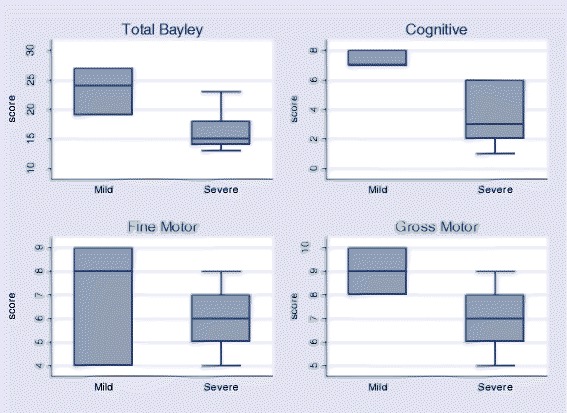
Total (summated cognitive, fine motor and gross motor) Bayley III-VN Scores and individual domain components for cases by severity of disease

**Fig. 4:**
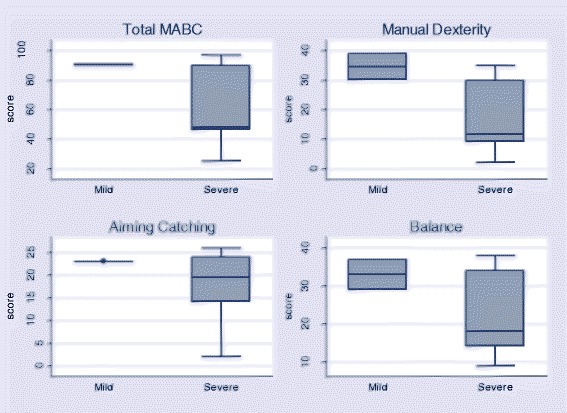
Total (summated manual dexterity, aiming catching and balance) Movement Assessment Battery for Children scores and individual domain components for cases by severity of disease

Comparing neurodevelopmental outcomes between the severity groups among children assessed using the Bayley III-VN, the five infants with severe disease had lower scores than the 3 infants who did not (*p* = 0.05). Median scores were also reduced in the individual domains in severe cases compared to mild, although only the cognitive domain reached statistical significance (Fig. 3, median 3 (IQR 2–6) compared with 7 (IQR 7–8) *p* = 0.02). Among older children, Movement Assessment Battery for Children scores were all reduced in those who had suffered severe disease (6 children) but only 2 cases were classified as mild and none of the differences observed were statistically significant (Fig. 4).

## Discussion

Current knowledge of outcome in neonatal tetanus is confined to the few studies of infants treated more than 25 years ago, and to our knowledge there are no data on outcome from settings with more developed critical care facilities. This study provides such data on a group of 17 survivors of neonatal tetanus treated in a specialist intensive care unit. The previous studies on outcome following neonatal tetanus were performed in settings without mechanical ventilation, with mortality rates ranging from 31 to 68% [5–7], while in our study infants had access to mechanical ventilation and the in-hospital mortality rate during the study period was only 9%. Our study shows that this improvement in hospital mortality was not offset by a significant increase in long-term disability in survivors, compared to previously published data.

Neurological abnormalities including cerebral palsy, epilepsy and coordination problems have previously been reported in 13–39% neonatal tetanus survivors [6, 7]. We did not find any evidence of these sequelae. This may be due to differences in management such as access to mechanical ventilation and continuous oxygen saturation and blood pressure monitoring minimizing the likelihood of hypoxic insults in our cohort. The only neurological abnormalities we detected in this study were the two cases of hearing impairment (12% survivors) and one case of mild motor abnormality. Perceptive hearing loss associated with tetanus was first reported in 1936 [14] in a 14 year-old boy with very mild tetanus treated with equine antitoxin intrathecally and intravenously. Deafness developed together with urticaria and was thought to be due to neuritis associated with serum sickness secondary to the use of heterologous antitoxin. Three further cases of perceptive deafness have been reported in individuals treated prophylactically for tetanus with equine antitoxin. In all three cases deafness followed clinical signs of serum sickness [15]. More recently perceptive deafness was described in a survivor of neonatal tetanus treated with human antitoxin without any signs of serum sickness and was attributed to a direct effect of tetanus toxin itself [16].

In specific studies of outcome in neonatal tetanus, deafness has not previously been reported. Anlar’s study of 24 neonatal tetanus survivors, all treated with equine antitoxin (two with additional intrathecal human antitoxin), specifically screened infants for hearing impairment but did not report any abnormalities [6]. Teknetzi carried out specialist audiology assessment in 38 children but did not note any deficit. Similarly Barlow also failed to report any abnormality in 23 children, none of whom were treated with antitoxin [7]. Whilst the cause of hearing impairment in our two cases is not clear, therapeutic drug monitoring is not available in our hospital and both infants received equine antitoxin and multiple courses of aminoglycoside and glycopeptide antibiotics for the treatment of nosocomial infections, either of which may have contributed.

Although total neurodevelopment scores and component domains showed a trend towards lower values in neonatal tetanus survivors compared to controls, no differences were statistically significant. The children in our study were between 25 and 69 months of age, thus two different tools were required to assess neurodevelopment, reducing statistical power. Nevertheless the absolute differences in scores between neonatal tetanus survivors and controls were relatively small and the relevance of these are not clear. Children in our cohort were also younger than those described in other studies and we do not know how their neurodevelopment will progress over time.

This is the first study to show a relationship between disease severity and subsequent neurodevelopmental outcome, with patients with more severe disease having reduced neurodevelopmental scores compared to those with mild disease. The only previous observation on this was from infants treated in Greece between 1966 and 1977, where Teknetzi reported that all four cases who showed ‘significant defects’ at follow-up had severe tetanus associated with periods of excessive spasms and cyanosis. In our study all neonates with severe tetanus received mechanical ventilation, experienced longer periods in hospital and increased incidence of nosocomial infection compared to those with mild disease, and any of these factors may have contributed to worse outcome in this group [1, 17, 18].

Our study has several limitations. We were unable to contact all cases discharged from hospital and therefore our sample may be subject to bias. However given the remote location of most cases low follow up rates are to be expected. Even in higher-income settings rates remain similar: Anlar reported that 70 out of 94 patients could not be contacted [6] and Teknezi was able to recruit only 38 out of 50 eligible children [6]. Matching these cases with controls was also challenging as children came from a variety of ethnic backgrounds and separate geographical locations. Whilst all children came from families of the same ethnic group, were of similar socioeconomic status and age and lived in the same village, other confounding effects are possible. Furthermore we were not able to blind assessors to the status of the children as many were familiar with cases from their stay in hospital. Our aim in carrying out this study was to ascertain whether improved survival rates of infants with neonatal tetanus treated at our hospital were associated with long-term sequelae. However in the remote and resource-limited settings where most neonatal tetanus occurs, identification and further management of these sequelae also presents us with substantial challenges due to the extremely limited availability of rehabilitation services outside major cities. Increasing international recognition of the importance of early childhood development and commitment to achieving the Sustainable Development Goals in many low and middle income countries means that this situation may be changing, and in future communities will be better placed to assist children at risk of poor development [8].

## Conclusions

The results from our study show that even with good intensive care facilities, neonatal tetanus still carries long-term consequences. Yet neonatal tetanus is a completely preventable disease and therefore efforts should be made to improve vaccination coverage and antenatal care, ensuring that prevention rather than treatment remains the priority.

## Abbreviations

Bayley III-VN: Bayley Scales of Infant and Toddler development (3rd edition) for Vietnam
IQR: Interquartile range
MABC: The Movement assessment battery for children
